# HLA-DPB1 genotype variants predict DP molecule cell surface expression and DP donor specific antibody binding capacity

**DOI:** 10.3389/fimmu.2023.1328533

**Published:** 2024-01-11

**Authors:** Yuxin Yin, Nwe Nwe Soe, Nicole M. Valenzuela, Elaine F. Reed, Qiuheng Zhang

**Affiliations:** ^1^ UCLA Immunogenetics Center, Department of Pathology & Laboratory Medicine, Los Angeles, CA, United States; ^2^ Department of Pathology, AdventHealth Tissue Typing Laboratory, Orlando, FL, United States

**Keywords:** transplantation, hematopoietic stem cell transplantation, HLA-DPB1, rs9277534, crossmatch, long non-coding RNA

## Abstract

The contribution of alloresponses to mismatched HLA-DP in solid organ transplantation and hematopoietic stem cell transplantation (HCT) has been well documented. Exploring the regulatory mechanisms of DPB1 alleles has become an important question to be answered. In this study, our initial investigation focused on examining the correlation between the rs9277534G/A SNP and DPB1 mRNA expression. The result showed that there was a significant increase in DPB1 mRNA expression in B lymphoblastoid cell lines (BLCLs) with the rs9277534GG genotype compared to rs9277534AA genotype. In addition, B cells with the rs9277534GG exhibited significantly higher DP protein expression than those carrying the rs9277534AA genotype in primary B cells. Furthermore, we observed a significant upregulation of DP expression in B cells following treatment with Interleukin 13 (IL-13) compared to untreated B cells carrying rs9277534GG-linked DPB1 alleles. Fluorescence *in situ* hybridization (FISH) analysis of DPB1 in BLCL demonstrated significant differences in both the cytoplasmic (p=0.0003) and nuclear (p=0.0001) localization of DP mRNA expression comparing DPB1*04:01 (rs9277534AA) and DPB1*05:01 (rs9277534GG) homozygous cells. The study of the correlation between differential DPB1 expression and long non-coding RNAs (lncRNAs) showed that lnc-HLA-DPB1-13:1 is strongly associated with DP expression (r=0.85), suggesting the potential involvement of lncRNA in regulating DP expression. The correlation of DP donor specific antibody (DSA) with B cell flow crossmatch (B-FCXM) results showed a better linear correlation of DP DSA against GG and AG donor cells (R^2^ = 0.4243, p=0.0025 and R^2^ = 0.6172, p=0.0003, respectively), compared to DSA against AA donor cells (R^2^ = 0.0649, p=0.4244). This explained why strong DP DSA with a low expression DP leads to negative B-FCXM. In conclusion, this study provides evidence supporting the involvement of lncRNA in modulating HLA-DP expression, shedding lights on the intricate regulatory mechanisms of DP, particularly under inflammatory conditions in transplantation.

## 1 Introduction

The human leukocyte antigen (HLA) is a complex genetic system consisting of highly polymorphic class I genes (HLA-A, -B, -C) and class II genes (HLA-DRB1/3/4/5, -DQA1, -DQB1, -DPA1, and -DPB1) (1, 2). These genes encode proteins that play a crucial role in immune response by facilitating the presentation of foreign antigens to immune cells and regulating the immune system’s recognition and response to pathogens (3). It is widely accepted that HLA donor specific antibodies (DSAs) are associated with antibody mediated rejection (AMR) and graft loss in solid organ transplantation (4). For over four decades, crossmatching between donor lymphocytes and recipient serum has been used to assess the histocompatibility of a particular donor/recipient pair prior to kidney, heart, and lung transplantation (5). However, prospective physical crossmatch limits the donor pool and results in long cold ischemia times.

The development of the Luminex single antigen bead (SAB) test has revolutionized the field. This test measures the binding of HLA antibodies in patient sera and provides intensities of HLA antibody binding to beads coated with HLA antigens (mean fluorescence intensity-MFI) that correlates with the antibody concentration. It allows characterization of HLA antibodies with high sensitivity and specificity to accurately predicting the presence or absence of DSA in a patient. The American Society for Histocompatibility and Immunogenetics defines virtual crossmatch (VXM) test as an assessment of immunologic compatibility based on patient’s HLA antibody profile compared with donor’s histocompatibility antigens. The VXM has increased the number of import donors and reduced the wait time and mortality in solid organ transplantation (6, 7). The sensitivity and specificity of a VXM in correlation with physical T and B lymphocyte crossmatches is high for patients carrying HLA-A, -B, -DR and -DQ DSA (8). However, Locker et al., showed that the accuracy of a VXM prediction for HLA-DP DSA on B lymphocytes is poor, likely due to variable expression of DP antigens on B cells (9). Similarly, Daniels et al., showed that strong HLA-DP DSA were not always correlated with positive B cell flow crossmatch (B-FCXM) results (10). HLA-DP has been considered to be less immunogenic than other HLA antigens, because of the low expression level. Early studies suggested the presence of anti-HLA-DP DSA does not reduce allograft survival in primary kidney transplant recipients (11). However, recent publications indicate the level of HLA-DP antibodies correlates with the strength of alloimmune responses in renal transplant patients (10, 12–16). Seitz et al., studied 1355 adult renal transplant recipients, and among them, 23 patients had preformed HLA DSA to DP (17). They demonstrated that patients with DP DSA had a significantly increased risk of AMR and reduced patient survival compared with the patient without HLA DSA. However, no correlation of DP DSA MFI with B-FCXM was found and the pre-transplant B-FCXM did not help inform on the risk of graft failure or AMR in patients with preformed DP DSA (17). These dis-concordance of DP DSA level with B-FCXM could be due to the differential expression of DP, which resulted in conflicting reports regarding the clinical relevance of HLA DSA to DP in solid organ transplantation (18–20).

The presence of pre-transplant HLA DSA limits engraftment of the stem cells and is associated with rates of graft failure in mismatched unrelated and hematopoietic stem cell transplantation (HCT) (21). Among matched unrelated donor (MUD) transplants, Ciurea et al. evaluated the impact of DSA in a cohort of 592 consecutive patients at M.D. Anderson Cancer Center. Anti-HLA antibodies were detected in 116 patients (19.6%), including 20 patients (3.4%) with anti-DP antibodies. Among the eight patients had HLA DSA against DP, three patients had engraftment failure compared to 16 of 584 (2.7%) patients without anti-HLA antibodies (22). In unrelated HCT, Spellman et al. found that 24% of the patient with engraftment failure had DSA against HLA-A, -B, or -DP, compared with 1% of the control group. Importantly, antibodies against HLA-DP DSA were present in 60% of engraftment failure cases (23). Among 567 patients who had HCT from cord blood, Jo et al., recently reported nine patients displayed pre-transplant HLA DSA to DP (n=5) or DQ (n=4). They found that DSAs against DP or DQ were associated with a significantly lower neutrophil and platelet engraftment rates, compared with patients without HLA DSA (24).

The expression of HLA-DP has been reported to linked to the rs9277534 single nucleotide polymorphism (SNP) located in the 3’ untranslated region (UTR). Specifically, the rs9277534A allele is associated with low expression, while the rs9277534G allele is correlated with high expression (25, 26). Since 3’UTR is not routinely performed at clinical testing, the study of > 32,000 European Caucasian samples showed > 99.6% concordance of the rs9277534 SNP with exon 3 sequences of DPB1 recently (27), which was identified as rs1126537, rs1126541, rs1042187, rs1042212, rs1042331, rs104335, and rs1071597 (ACCACTC or GTTGTCT haplotypes) in a later study (28). The importance of the DP expression in HCT was first demonstrated by Petersdorf et al., and the rs9277534-DPB1 haplotypes were defined by genotyping rs9277534 in a cohort of 3505 patients. They found that among recipients who received transplants from donors with rs9277534A-linked DPB1 mismatches, the risk of grades II to IV acute Graft-versus-host disease (GVHD) was higher for recipients with rs9277534G-linked DPB1 mismatches than for recipients with rs9277534A-linked DPB1 mismatches (26). These findings emphasize the importance of considering HLA-DPB1 expression levels when selecting HLA-DPB1 mismatched unrelated donors for HCT (29, 30). In addition, Monos et al., showed that patients carrying low expression rs9277534-AA alleles that received donor with GX (X = A or G) alleles had significantly increased risk in developing *de novo* DP DSA in pediatric solid organ transplants (31). However, the mechanistic understanding of the rs9277534 SNP on HLA-DP expression remains elusive, and its clinical significance in transplantation requires further investigation.

Numerous studies have been conducted to understand the regulatory mechanisms of HLA-DP expression. The genomic structure of HLA-DPB1 encompasses 11.28 kb with a transcript length of 1.56 kb organized in five coding exons and a sixth exon in the 3′ UTR (32). However, it remains unclear whether the rs9277534 SNP serves solely as a marker, or if it regulates HLA-DPB1 expression. Interestingly, both high and low expressed DPB1 alleles share the same promoter sequences, and studies have shown that the difference of DP expression can be detected at the mRNA level (26). This suggests that post-transcriptional regulation may play a role in HLA-DP expression. Long non-coding RNAs (lncRNAs) are RNA molecules transcribed from DNA that do not code for proteins but play crucial roles in regulating gene expression (33). Recent studies have highlighted the involvement of lncRNAs in the regulation of immune responses, particular in relation to HLA gene expression. For instance, IncRNA HCP5 has been associated with HLA-B expression (34), while IncRNA HOXTAIR has been shown to promote HLA-G expression by inhibiting miRNA-152 in gastric center (35). Based on these findings, we hypothesized that lncRNAs might also play a role in regulating HLA-DPB1 expression. HLA Class I molecules are constitutively expressed by all nucleated cells, while HLA class II molecules are primarily expressed by antigen-presenting cells, such as dendritic, macrophage, and monocyte cells and B lymphocytes. Under inflammatory conditions, such as during transplantation, HLA class II expression levels can be upregulated in endothelial cells by proinflammatory cytokines, thereby contributing to transplant rejection (36). With this in mind, we investigated the cell surface expression of DPB1 alleles linked to the rs9277534A and rs9277534G in B lymphocytes with and without inflammatory cytokine stimulation. To further explore the potential differences between rs9277534A and rs9277534G linked DPB1, we performed Fluorescence *in situ* hybridization (FISH) to examine the molecular localization of DPB1 mRNA. Lastly, we tested our hypothesis that the DPB1 expression could be regulated by lncRNA.

## 2 Results

### 2.1 Differential DPB1 mRNA expression according to rs9277534 SNP in B lymphoblastoid cell lines (BLCLs)

**Figure 1:**
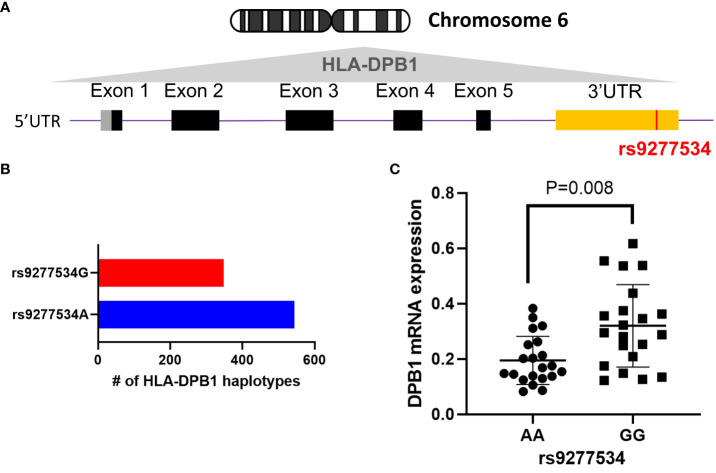
The rs9277534 SNP A or G is linked HLA-DPB1. **(A)** The schematic representation of the HLA-DPB1 allele within Chromosome 6 HLA region (6p21.1-21.3) is shown. The bottom panel illustrates DPB1 genomic structure, with black boxes denoting coding exons, gray and yellow boxes indicating 5’ and 3’ UTR, respectively. The purple line connecting the boxes represents introns, and the SNP (rs9277534, located in the 3’UTR) is highlighted in red. **(B)** The number of full genomic HLA-DPB1 haplotypes, containing the rs9277534 SNP, from IMGT/HLA database v3.54. rs9277534 has two groups (rs9277534A and rs9277534G) linked to HLA-DPB1. **(C)** Examination of the HLA DPB1 expression in 14 BLCLs. Both alleles linked to either rs9277534A or rs9277534G were used for each cell line. HLA-DPB1 mRNA expression was determined through a qRT-PCR assay with experiments involving triplicates. GAPDH served as endogenous control, and the scatter dot plot displays mean standard deviation (SD).

HLA-DPB1 genes display extensive polymorphism, with a current total of 892 identified full genomic alleles, each containing the rs9277534 SNP, out of 2393 alleles according to the most recent HLA/IMGT database v3.54 (37). Among these, 544 DPB1 alleles are linked to rs9277534A, while 348 allelic DPB1 variants are linked to rs9277534G (**Figures 1A, B**). To investigate the association of rs9277534 SNP with DPB1 mRNA expression levels, we conducted quantitative real-time PCR (qRT-PCR) to measure DPB1 mRNA expression in BLCLs. Our findings demonstrated a significantly higher expression of DPB1 mRNA in the rs9277534GG group (n=7, with triplicate measurements for each sample) compared to the rs9277534AA group (n=7, with triplicate measurements for each sample) in BLCLs (p=0.008, **Figure 1C**). DPB1 alleles covered in the 14 BLCLs are listed in **Supplementary Table 1**.

### 2.2 Correlation between DP DSA and B-FCXM based on the rs9277534 SNP

**Figure 2:**
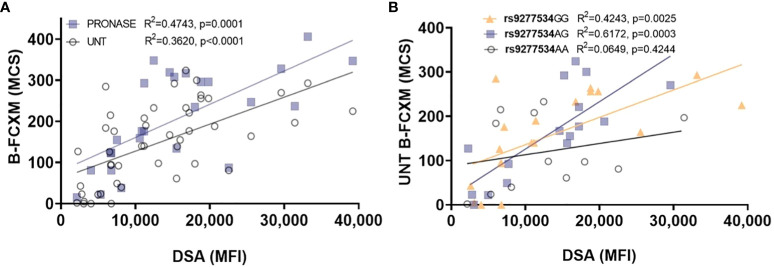
Examining the correlation between DP DSA and B-FCXM. **(A)** The correlation of DP DSA MFI on B-FCXM with and without pronase (UNT) treatment. **(B)** A robust linear correlation in B-FCXM with DP DSA against rs9277534GG and AG donor cells, whereas such correlation is not observed in cells with rs9277534AA. MCS, median channel shift.

The accuracy of B-FCXM is often compromised by the binding of non-HLA antibodies to B cells in peripheral blood mononuclear cells (PBMCs), including auto antibodies and treatment of rituximab (anti-CD20). Pronase, a proteolytic enzyme, can effectively remove Fc receptors and CD20 from the B cell surface, enhancing the specificity and sensitivity of the assay. The correlation between DP DSA (**Supplementary Table 2**) with B-FCXM results were investigated both with and without pronase treatment. In **Figure 2A**, the correlation of the sum of DP DSA MFI on B-FCXM is depicted for samples with pronase treatment (n=26) and without pronase treatment (n=47). The correlation between DP DSA and untreated B-FCXM was found to be poor, with an R^2^ of 0.3620 (p<0.0001). However, a significantly improved linear correction was observed between B-FCXM and MFI of the sum of DP DSA with pronase treatment (R^2^ = 0.4743, p=0.0001). Further analysis explored the correlation of DP SNPs with untreated B-FCXM results. A superior linear correlation was found in untreated B-FCXM with DP DSA against rs9277534GG (R^2^ = 0.4243, p=0.0025) and 9277534AG (R^2^ = 0.6172, p=0.0003) donor cells, compared to cells with 9277534AA DP genotypes (R^2^ = 0.0649, p=0.4244) (**Figure 2B**).

### 2.3 DP cell surface expression in B cells of PBMCs stimulated with Interleukin 13 (IL-13) cytokine

**Figure 3:**
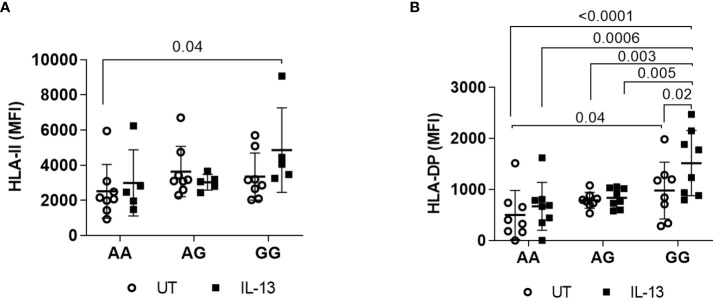
Induction of cell surface expression by IL-13 cytokine. **(A)** IL-13 induced HLA class II cell surface expression. PBMCs were cultured with IL-13 (100ng/mL) for 48 hours, and cells stained with anti HLA-II antibodies were gated out of PBMCs. **(B)** IL-13 induced HLA DP cell surface expression on PBMCs. The combinations of AA, AG, and GG represent the two DPB1 alleles present in each donor cell, linked to the rs9277534 SNP. Only p values of < 0.05, considered statistically significant, are marked. The scatter dot plot presents mean SD.

To simulate the inflammatory conditions relevant to transplant settings, B cells within PBMCs were subjected to IL-13 treatment, as BLCLs lack IL-13 receptors (38). IL-4 was not employed since it stimulates both T cells and B cells (39–41). Overall, no significant differences were observed in total HLA-class II expression among B cells from healthy volunteer donors with rs9277534AA (n=8), rs9277534AG (n=8), and rs9277534GG (n=8), both with and without IL-13 treatment. However, a noteworthy finding emerged when comparing IL-13 treated B cells carrying rs9277534GG, which exhibited a significant difference compared to untreated B cells with rs9277534AA (p=0.04, **Figure 3A**). Additionally, a significant difference in DP expression on untreated B cells was noted when comparing rs9277534GG and rs9277534AA (p=0.04, **Figure 3B**). Interestingly, IL-13 treatment led to a substantial increase in DP expression specifically in B cells carrying rs9277534GG-linked DP alleles (p=0.02), with no such effect observed in B cells with rs9277534AA or rs9277534AG-linked DP alleles. Moreover, when treated with IL-13, the expression of DP was significantly higher in the rs9277534GG subgroup compared to rs9277534AA (p=0.0006) or rs9277534AG-linked DP (p=0.005).

### 2.4 Cellular localization of DPB1 mRNA in BLCLs

**Figure 4:**
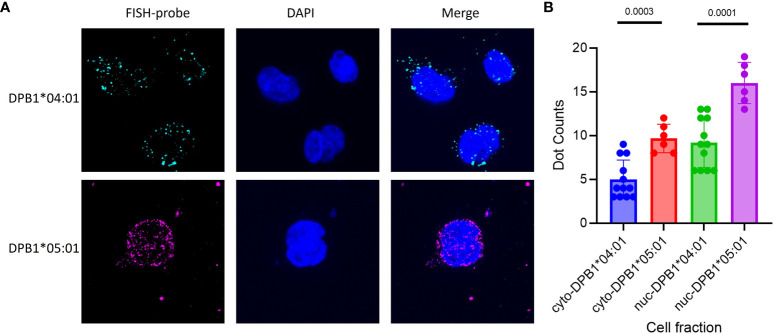
FISH of DPB1 was conducted on BLCLs to assess the expression of DPB1 mRNA in cellular compartments. Two high-frequency HLA-DPB1 alleles were chosen for the primary test, with one BLCL being homozygous for DPB1*04:01 linked to rs9277534AA and the other homozygous for DPB1*05:01 linked to rs9277534GG. **(A)** Representative RNA FISH images of DPB1*04:01 (Cyan) and DPB1*05:01 (Magenta) RNA in BLCLs, with DAPI staining the nucleus (Blue). **(B)** The Box plot showing cytoplasmic and nuclear FISH counts for DPB1*04:01 and DPB1*05:01. The prefixes “cyto-” and “nuc-” denote the cytoplasmic and nuclear compartments, respectively, within a cell fraction.

To precisely determine the cellular localization of HLA-DPB1 mRNA, FISH was conducted, comparing BLCLs with rs9277534AA genotype (DPB1*04:01 homozygous) and rs9277534GG genotype (DPB1*05:01 homozygous). Each set of single-stranded DPB1*04:01-exonic (42 oligos per set) and DPB1*05:01-exonic (35 oligos per set) oligos complementary to the target genes, was labeled accordingly (**Figure 4A**, **Supplementary Table 3**). The findings revealed a significant increase in mRNA expression for the rs9277534GG genotype (DPB1*05:01 homozygous) compared to the rs9277534AA genotype (DPB1*04:01 homozygous) in both cytoplasmic (p=0.0003) and nuclear compartments (p=0.0001, **Figure 4B**).

### 2.5 Correlation between lncRNA and DPB1 mRNA expression

**Figure 5:**
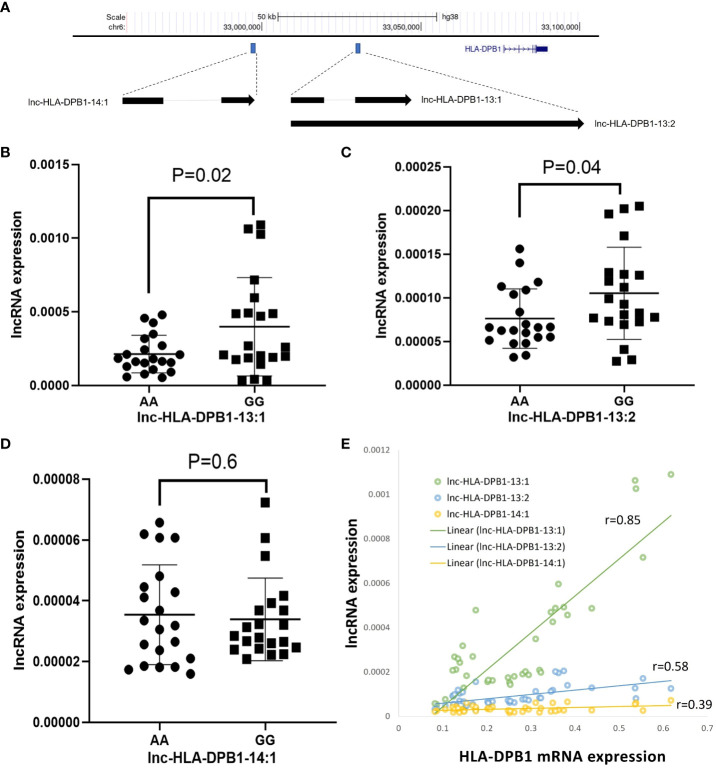
Exploring the potential involvement of lncRNAs in HLA-DPB1 regulation. **(A)** A schematic representation illustrates the genomic location of lncRNAs (lnc-HLA-DPB1-13:1, lnc-HLA-DPB1-13:2, and lnc-HLA-DPB1-14:1) in relation to HLA-DPB1 on Chromosome 6 (hg38). The blue box highlights the location of lncRNAs, and the blue gene structure represents HLA-DPB1. The arrow indicates the direction of the lncRNA transcript, and splicing is denoted by a light gray line. **(B–D)** Comparative analysis of lncRNA expression between rs9277534A and G-linked HLA-DPB1. Experiments were conducted in triplicates, with GAPDH as the endogenous control. The scatter dot plot displays mean SD. **(E)** The correlation between lncRNA expression and HLA-DPB1. The correlation coefficient ranges between 0.8 and 1, indicating a very strong positive association, while a coefficient between 0.2 and 0.6 suggests a moderate to weak positive association.

To explore the potential regulatory mechanisms governing DP expression, we conducted qRT-PCR analysis to examine the correlation between DPB1 mRNA and specific lncRNAs in BLCLs. Our investigation identified three candidate lncRNAs, namely lnc-HLA-DPB1-13:1, lnc-HLA-DPB1-13:2, and lnc-HLA-DPB1-14:1, positioned upstream of the HLA-DPB1 gene (**Figure 5A**). Notably, both lnc-HLA-DPB1-13:1 and lnc-HLA-DPB1-13:2 share the same genomic location but exhibit distinct alternative splicing patterns, while lnc-HLA-DPB1-14:1 is situated farther away from lnc-HLA-DPB1-13:1 and lnc-HLA-DPB1-13:2 (**Figure 5A**).

As shown by qRT-PCR experiments, the expression of lnc-HLA-DPB1-13:1 RNA (p=0.02; **Figure 5B**) and lnc-HLA-DPB1-13:2 RNA (p=0.04; **Figure 5C**) was significantly higher in BLCLs carrying the rs9277534GG genotype compared to the rs9277534AA genotype. However, no significant difference in the expression of lnc-HLA DPB1-14:1 was observed in BLCLs (p=0.6, **Figure 5D**). Furthermore, a robust correlation was identified between lnc-HLA-DPB1-13:1 and DPB1 mRNA expression (r=0.85, p<0.0001, **Figure 5E**), whereas no such correlation was observed for lnc-HLA-DPB1-13:2 and lnc-HLA-DPB1-14:1. These findings imply a potential pivotal role of lnc-HLA-DPB1-13:1 in the molecular regulation of mRNA expression of DPB1.

## 3 Discussion

The importance of HLA-DPB1 in HCT and solid organ transplantation has been highlighted in recent studies (10, 13–15, 26). Thomas et al. first reported a correlation between differential DP expression and the rs9277534 variant, which was associated with Hepatitis B virus recovery (25). They demonstrated that individuals with the homozygous rs9277534G genotype exhibited higher mRNA levels and DP expression in peripheral blood leukocytes. Subsequently, Petersdorf et al. showed that rs9277534G variant at the 3’UTR of HLA-DPB1 was associated with increased HLA-DPB1 expression (42). They also found that patients with rs9277534A genotype who received HCT from donors with rs9277534A-linked DPB1, a significantly increased risk of acute GVHD was observed in patient with rs927753G-linked DPB1 mismatches compared to patients who received donors carrying rs9277534A-linked DPB1 mismatches (26). However, the regulatory mechanism underlying the differential expression of DPB1 remains unknown.

Previous studies have implicated post-transcriptional regulation of HLA genes, such as HLA-C, through miRNA (43). However, recent studies did not find evidence of regulation of DPB1 expression at thers9277534 SNP through miRNA (44, 45). Recently, Thomas et al. showed the overexpression of IncRNA GHSROS induced the downregulation of HLA-DRA, HLA-DPB1, HLA-DPA1, and HLA-DRB3 expression in breast cancer cells, indicating the potential role of IncRNA in regulation of major histocompatibility complex (MHC). However, the study does not provide explanation of differential DPB1 expression with different SNP (46). Our results showed significant differences in both the cytoplasmic and nuclear localization of DPB1 mRNA expression comparing DPB1*04:01 (rs9277534AA) and DPB1*05:01 (rs9277534GG) homozygous cells. These findings suggest that the regulation of DP expression possibly initiates in the nuclear. In our investigation, we identified lnc-HLA-DPB1-13:1, but not lnc-HLA-DPB1-13-2 and lnc-HLA-DPB1-14-1, has a strong correlation with HLA-DPB1 mRNA expression. As we are aware, the regulation of lncRNA is intricate (47), and they exert diverse functions, including acting as “transcriptional enhancers” or “transcriptional silencers,” modifying chromatin structure near the target gene’s promoter and influencing the biding of transcription factors and RNA polymerase (48, 49); lncRNAs can control the processing and stability of other RNA molecules, impacting splicing, polyadenylation, and other processing events (50, 51). The observed strong positive correlation between lnc-HLA-DPB1-13:1 and DPB1 mRNA expression implies a regulatory mechanism resembling transcriptional enhancement. These findings provide valuable insights into the molecular mechanisms underlying the regulation of HLA-DPB1 expression, which may have important implications for understanding transplant rejection and autoimmune diseases. One limitation of this study is the lack of a suitable inhibitor for lnc-HLA-DPB1-13:1, attributed to its short sequence and its status as an alternative splice variant of lnc-HLA-DPB1-13:2. Consequently, in-depth functional exploration of the lncRNA on specific rs9277534A or G linked DPB1 allele need to be further confirmed.

Since the rs9277534A DPB1 alleles are reported to be associated with low expression, while the rs9277534G DPB1 alleles are correlated with high expression (26). We investigated the impact of DP expression level on B-FCXM results. Our results showed a significantly better correlation of DP DSA strength with B-FCXM results with GG and AG donor cells (high expression) compared to AA donor cells (low expression). These results explain the controversial reports on lacking the correlation of strong DP DSA with positive B-FCXM, which were due to the low expression DP antigens carried by the donor cells (9). This also implies that the VXM prediction on rs9277534G-related donor cells would have a better correlation with B-FCXM. Organ transplantation involves varying degrees of surgical trauma, tissue damage, and ischemic reperfusion injury (IRI), which can trigger innate inflammatory responses (52). Notably, IL-13 levels were elevated in preoperative recipients and persisted in ischemia/reperfusion injury (IRI)+ recipients, indicating a potential involvement of adaptive immunity in the IRI process (53). Similarly, another research group reported inflammatory cell death, tissue damage, and mortality in Sars-Cov-2 infection and cytokine shock syndromes (54). Constitutive expression of HLA Class II molecules is restricted to antigen presenting cells but can be upregulated under inflammatory conditions. Our results showed a significantly increased DP expression in B cells with IL-13 treatment, suggesting a potential higher risk of worse outcomes in rs9277534G linked DPB1 donors. However, Meurer et al. demonstrated that overnight treatment of B cells with interferon-γ abrogated the expression difference of DP associated with rs9277534 SNP (45), therefore, DSA to low expression DP could exert an increased risk of AMR and graft loss, particularly under inflammatory conditions.

In conclusion, our study sheds light on the potential regulatory mechanisms of HLA-DPB1 through lncRNAs. Understanding the regulatory mechanisms may aid in identifying patients at a higher risk in both HCT and solid organ transplantation. Additionally, our study provides explanations for the variabilities in VXM predictions for patients with DP DSA, potentially enhancing the clinical understanding of solid organ transplantation. Further investigations of the interactions between lncRNA and DPB1 expression may lead to the discovery of novel therapeutic strategies. Understanding the mechanisms underlying cytokine-induced HLA-DPB1 expression may provide important insights into the development and progression of these diseases and could pave the way for the identification of novel therapeutic targets.

## 4 Materials and methods

For a comprehensive overview, refer to **Supplementary Figure 1**, which outlines the sequence of tests conducted on various cell types.

### 4.1 PBMCs isolation from donors

This study enrolled healthy donors from UCLA Immunogenetics Center and received approval from UCLA Institutional Review Board (IRB#10-001689). PMBCs were isolated from fresh whole blood using Ficoll-Paque (GE Healthcare) density gradient centrifugation method. In detail, whole blood was drawn into anti-coagulant acid citrate dextrose (ACD) tubes. The anticoagulated blood was diluted at a 1:1 ratio with phosphate buffered saline without calcium and magnesium (1X PBS). The diluted blood samples were overlaid on the top of Ficoll-Paque (1:1 ratio) and centrifuged at 2000 rpm for 20 minutes. The buffy coat layer obtained was mixed with 1X PBS and centrifuged at 800 rpm for 10 minutes to eliminate platelets. The resulting cell pellet was resuspended in RPMI culture medium supplemented with 10% FBS and 10μg Pen/Strep (ThermoFisher). Cell counting, using trypan blue and a hemocytometer, was performed, and the concentration was adjusted to 2.5 x10^6^ cells/mL.

### 4.2 IL-13 cytokine stimulation

PBMCs were plated in a 96 well plate at 0.5x10^6^ cells per 200μl concentration per well. IL-13 (100 ng/mL) (Sigma) were introduced to the cell culture. The cells were then incubated at 37°C with 5% CO2. Following 48 hours of IL-13 stimulation, the cells were harvested to assess the cell surface expression of HLA-DP molecule.

### 4.3 BLCLs culture conditions

Fourteen BLCLs underwent testing for HLA-DPB1 cell surface expression. The cell lines were cultured in cRPMI, composed of RPMI-1640 (GE Healthcare) supplemented with 10% FBS (Omega), 1% Amphotericin B (Corning), 1% Penicillin Streptomycin Solution (Corning). All cultures were maintained at 37°C in a 5% CO_2_ environment.

### 4.4 Measurement of HLA-DP cell surface expression

Flow cytometry analysis was employed to assess the expression of HLA-DP molecules on lymphocytes. IL-13 stimulated PBMCs were transferred into 1.5 mL microcentrifuge tubes and pelleted at 1500 rpm for 5 minutes. The cell pellets were resuspended with 100μl (1ug/mL concentration) of anti-HLA-DP antibodies (One lambda), anti-HLA Class-II antibodies (One lambda), or isotype control IgG (Biolegend), followed by a 20-minute incubation on ice. After washing with FACS buffer (1% FBS in 1X PBS) and centrifugation at 1500 rpm for 5 minutes, cells were incubated with fluorescein isothiocyanate (FITC)-conjugated anti-mouse secondary antibody (1:200), Cy5.5 conjugated CD3 antibody (1:40), and allophycocyanin (APC)-conjugated CD19 antibody (1:40) on ice in the dark for 20 minutes. Subsequent washing with FACS buffer and resuspension with 300μl FACS buffer preceded the measurement of cell surface expression of HLA-DP or HLA-Class II molecules using FACSCanto II flow cytometry (BD Biosciences). FITC-labeled lymphocytes were gated on CD3 or CD19 expressed, and FlowJo (TreeStar Inc) was used for data analysis.

### 4.5 Solid phase SAB assay

The specificity of HLA-DP antibodies or HLA-Class II antibodies utilized in the analysis of HLA molecule cell surface expression was assessed through a Luminex bead-based single antigen assay (One lambda). A mixture of 20μl of anti-HLA-DP or anti-HLA-Class II antibodies were incubated with 5μl of Luminex microbeads coated with purified HLA antigen in the dark for 30 minutes at room temperature (20-25°C). Following incubation, the beads underwent washing with wash buffer. Detection of HLA antibodies bound to the beads was achieved by adding anti-phycoerythrin (PE)-conjugated goat anti-mouse IgG antibody. The fluorescence emission of Luminex beads was then detected and quantified using Luminex 100/200 system (Luminex).

### 4.6 Positive sera selection for HLA DP antibodies

Positive sera of anti-HLA-DP antibodies were retrospectively chosen from patients exhibiting exclusive anti-HLA DP antibodies. HLA-antibodies within patients’ sera were assessed using previously mentioned SAB assay. In summary, 20μl of a patient’s sera and 5μl of Luminex microbeads were incubated for 30 minutes. As a secondary antibody, PE-conjugated goat anti-human IgG was employed. A normalized value exceeding 2000 MFI after normalization was deemed indicative of a positive result.

### 4.7 Lymphocyte flow cytometry crossmatch

Cryopreserved donor lymphocytes were thawed, washed, and resuspended in McCoy medium at a final concentration of 1 x 10^6^/mL. Twenty microliters of patient’s sera were added to the cell suspension and incubated for 60 minutes. The sample were then washed and subjected to additional incubation with CD3, CD19, and FITC conjugated antibodies. Data acquisition was performed using FACSCanto II flow cytometry (BD Biosciences).

### 4.8 Next generation sequencing for high resolution typing

Multiplex long-range PCR primers (One Lambda) were designed for the co-amplification of Class I (HLA-A, -B, -C) and Class II (HLA-DRB1, -DQB1, -DPB1). Following the equimolar pooling of Class I and Class II PCR products, the amplicons underwent library preparation using the TruSeq Nano DNA sample preparation kit (Illumina). Paired-end sequencing runs were conducted using MiSeq Reagent Kit v2, 500 cycles (Illumina). Data analysis is performed using Omixon HLA Twin Version 1.1.4 (Omixon).

### 4.9 Taqman^®^ Genotyping of rs9277534

The rs9277534 SNP genotyping was conducted using commercially available Taqman^®^ Genotyping Assays (Life Technologies). Reactions were prepared using TaqMan^®^ Genotyping Master Mix (Life Technologies), with a total input DNA of 10-20ng for each well. Two negative controls for each assay were included for quality control. The reaction plate was loaded onto the 7500 Fast System Real-Time PCR system to generate raw data. After completion, the data were analyzed using TaqMan^®^ Genotyper Software Version1.3.1 (Life Technologies).

### 4.10 Direct chromosomal phasing of HLA-DPB1 Exon 2 and rs9277534

A direct phasing method was devised to physically connect HLA-DPB1 exon 2 with rs9277534. Oligonucleotide probes specific to rs9277534A and rs9277534G were employed to distinguish between the two haplotypes, utilizing the HSE Haplotype Specific Extraction kit (Generation Biotech). The haploid DNA was genotyped for rs9277534 using Taqman^®^, and sequencing was performed for HLA-DPB1 exon 2.

### 4.11 Single molecule RNA FISH

FISH probes were enzymatically labeled with distinct spectrally fluorescent dyes using modified FISH probe labeling protocols (55). BLCLs were seeded on coverslips (Fisher Scientific) and cultured with cRPMI media in a 12-well plate. When cell density reached 70-80%, cells were washed once with PBS, fixated with 4% PFA, and permeabilized with 70% ethanol at 4°C overnight. Cells were rehydrated in 2xSSC buffer with 10% formamide and immersed in hybridization buffer (Biosearch) with a final probe concentration of 1 ng/μl for >16 hours. The cells were then washed twice with wash buffer A (Biosearch) for 30 minutes at 37°C. DAPI (Invitrogen) was added in the second wash to a final concentration of 0.5 ug/mL. Samples were washed once with wash buffer B (Biosearch) for 5 minutes. The coverslips were mounted on microscope slides in ProLong Gold antifade media (Invitrogen) overnight before microscopy. Cells were imaged on a Leica TCS SP8 light-sheet microscope equipped with a 63x, 1.4 Numerical Aperture (NA) oil-immersion objective (Leica) and a Leica sCMOS-camera. Images were acquired in ~200 nm z-dimension axis steps across ranges of approximately 4 μm for ES cells. Nuclei and cytoplasm were segmented by drawing outlines manually via DAPI and fluorescent dye signals, respectively. For image analysis, Multicolor z-stack images obtained from the Leica confocal SP8 were split into individual channels and imported into FISH-quant (56).

### 4.12 qRT-PCR for HLA-DPB1 and lncRNA expression

The lncRNA sequences for HLA-DPB1 were sourced from LNCipedia v5.2 (https://lncipedia.org/), revealing three identified transcripts for the lncRNA of HLA-DPB1 (lnc-HLA-DPB1-13:1, lnc-HLA-DPB1-13:2, and lnc-HLA-DPB1-14:1). Expression levels of HLA-DPB1 and lncRNAs (**Supplementary Table 4**) were assessed using qRT-PCR for 14 BLCLs. Total RNA isolation employed the RNeasy Plus Mini Kit (Qiagen), with reverse transcription conducted using the Maxima H Minus cDNA Synthesis Master Mix (ThermoFisher) as per the manufacturer’s protocol. qRT-PCR was performed for HLA-DPB1, lncRNA, and GAPDH (endogenous control) transcript levels using TaqMan^®^ Gene Expression assays (Life Technologies) on the ABI 7500 Real Time PCR system (Applied Biosystems). The data plots were presented as normalized individual data points (2^-deltaCt [delta Ct= Ct gene of interest-Ct endogenous control]).

### 4.13 Statistical analysis

Each qRT-PCR or protein expression was conducted in triplicates. Statistical analysis employed the Student’s *t* test or ANOVA using GraphPad Prism 9 (GraphPad Software, Inc.). Linear regression analysis and Pearson’s squared correlation coefficients (R^2^) were obtained Graphpad Prism 9. P values less than 0.05 were deemed statistically significant.

## Data availability statement

The original contributions presented in the study are included in the article/**Supplementary Material**. Further inquiries can be directed to the corresponding author.

## Ethics statement

The studies involving humans were approved by UCLA Institutional Review Board (IRB#10-001689). The studies were conducted in accordance with the local legislation and institutional requirements. The participants provided their written informed consent to participate in this study.

## Author contributions

YY: Conceptualization, Data curation, Formal Analysis, Investigation, Methodology, Project administration, Resources, Software, Supervision, Validation, Visualization, Writing – original draft, Writing – review & editing. NS: Data curation, Methodology, Writing – review & editing. NV: Data curation, Resources, Writing – review & editing. ER: Conceptualization, Resources, Supervision, Writing – review & editing. QZ: Conceptualization, Project administration, Resources, Supervision, Writing – original draft, Writing – review & editing.
