# Supplementary material for: HLA-DPB1 genotype variants predict DP molecule cell surface expression and DP donor specific antibody binding capacity
Source: Front Immunol. 2024 Jan 11;14:1328533. doi: 10.3389/fimmu.2023.1328533 (PMC10808447; doi:10.3389/fimmu.2023.1328533)
Supplement: Supplementary file 1 [file Presentation_1.pptx]

## Slide 1
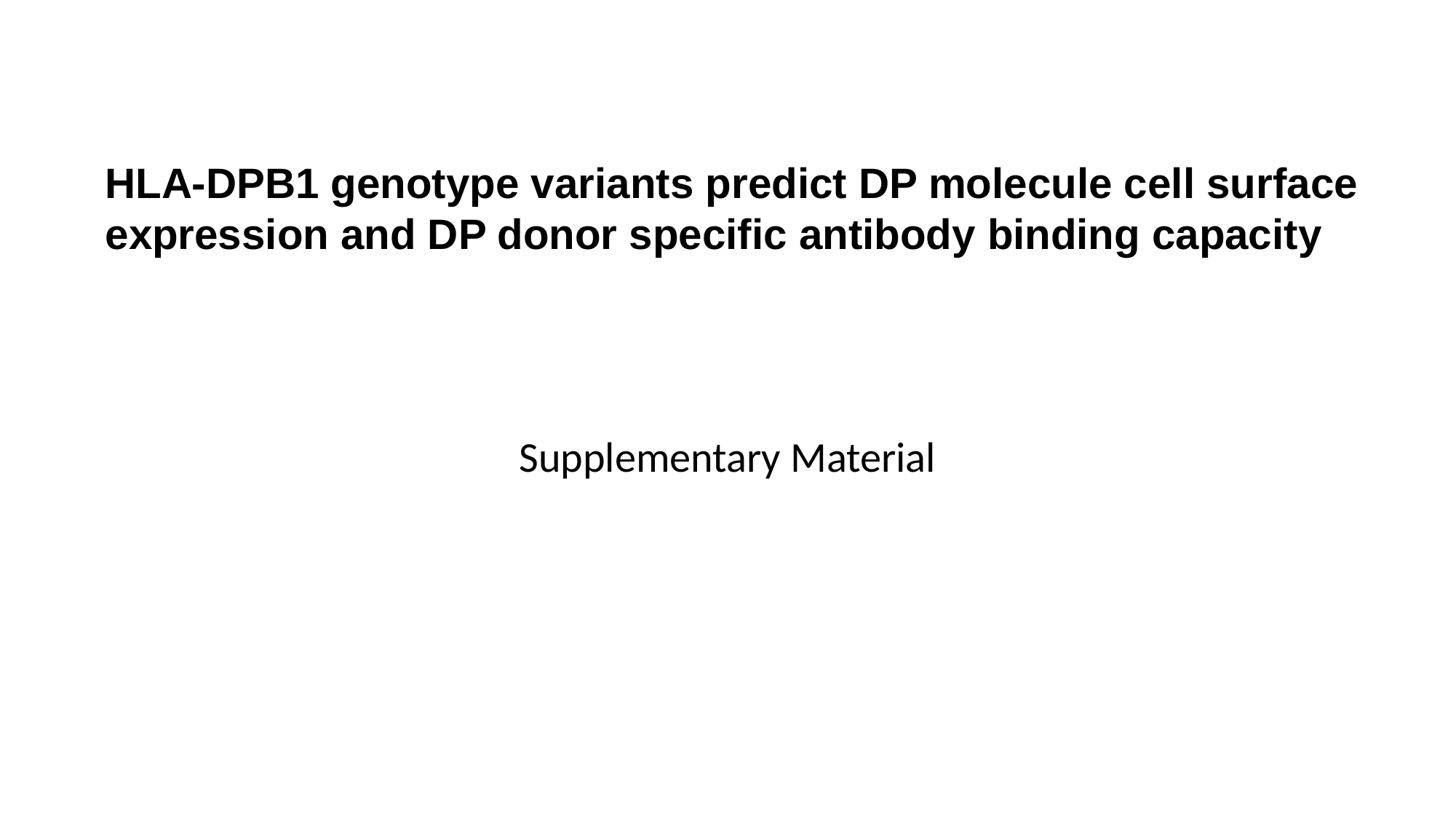

HLA-DPB1 genotype variants predict DP molecule cell surface expression and DP donor specific antibody binding capacity
# Supplementary Material

## Slide 2
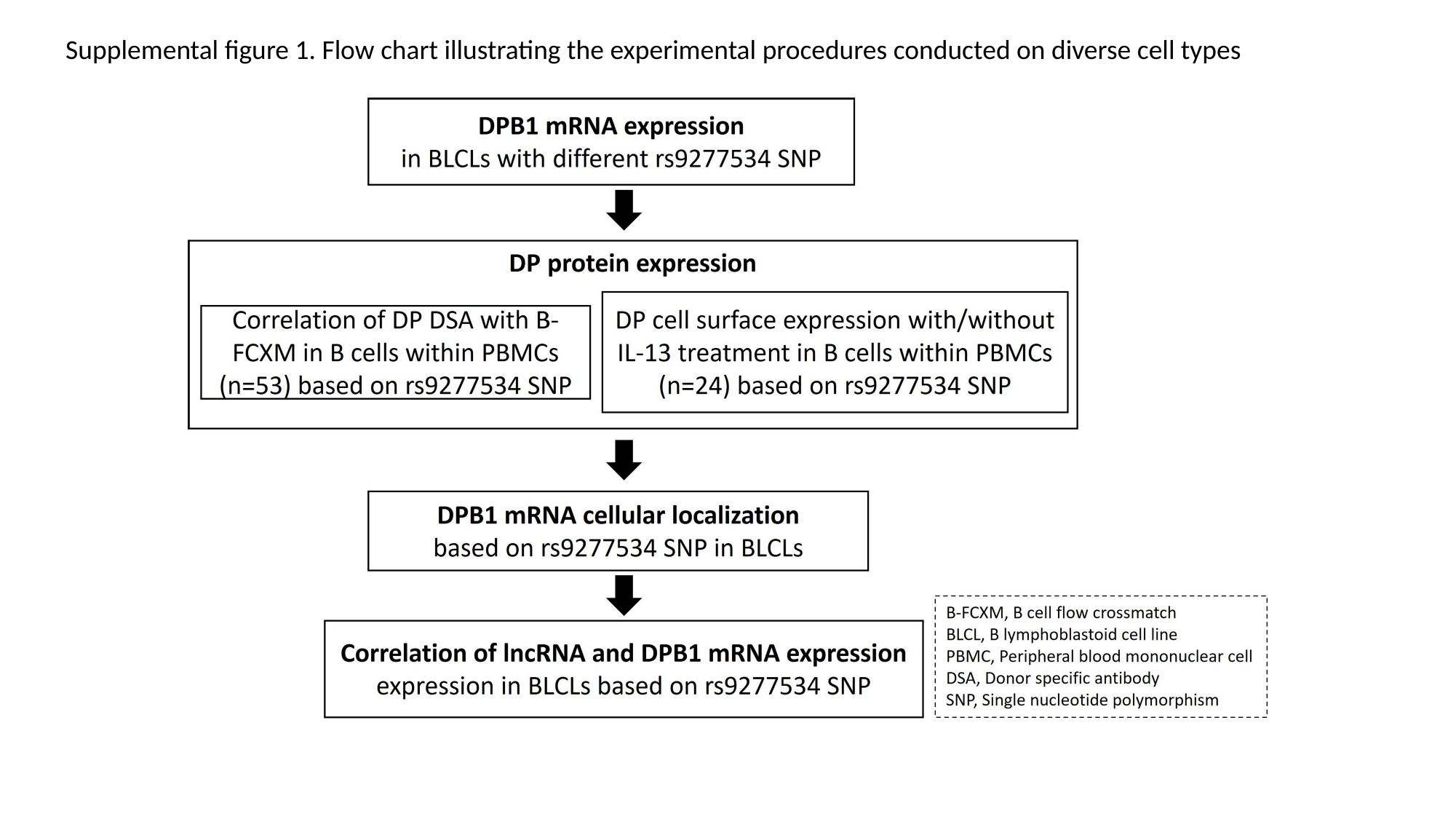

Supplemental figure 1. Flow chart illustrating the experimental procedures conducted on diverse cell types

## Slide 3
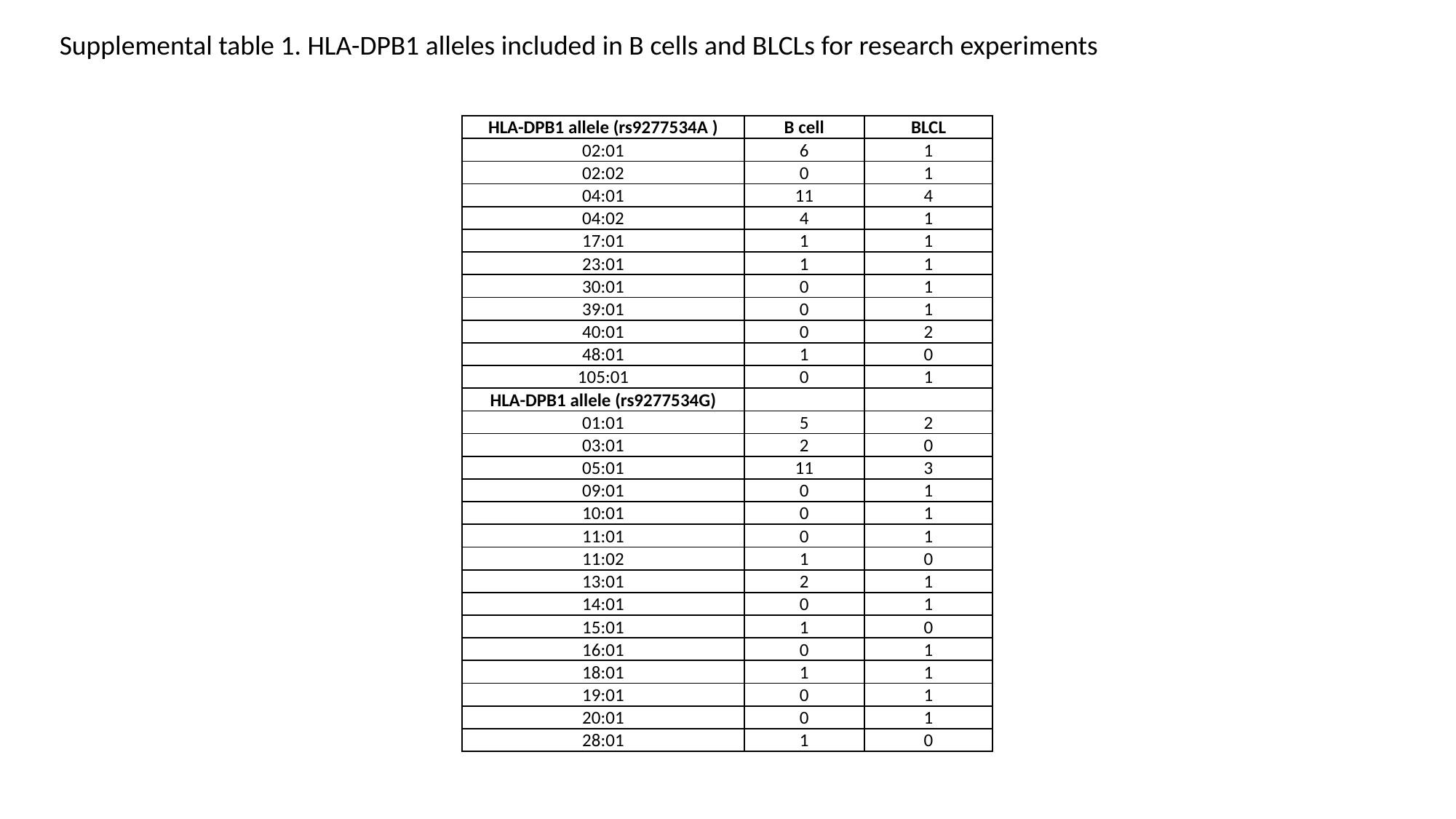

Supplemental table 1. HLA-DPB1 alleles included in B cells and BLCLs for research experiments
| HLA-DPB1 allele (rs9277534A ) | B cell | BLCL |
| --- | --- | --- |
| 02:01 | 6 | 1 |
| 02:02 | 0 | 1 |
| 04:01 | 11 | 4 |
| 04:02 | 4 | 1 |
| 17:01 | 1 | 1 |
| 23:01 | 1 | 1 |
| 30:01 | 0 | 1 |
| 39:01 | 0 | 1 |
| 40:01 | 0 | 2 |
| 48:01 | 1 | 0 |
| 105:01 | 0 | 1 |
| HLA-DPB1 allele (rs9277534G) | | |
| 01:01 | 5 | 2 |
| 03:01 | 2 | 0 |
| 05:01 | 11 | 3 |
| 09:01 | 0 | 1 |
| 10:01 | 0 | 1 |
| 11:01 | 0 | 1 |
| 11:02 | 1 | 0 |
| 13:01 | 2 | 1 |
| 14:01 | 0 | 1 |
| 15:01 | 1 | 0 |
| 16:01 | 0 | 1 |
| 18:01 | 1 | 1 |
| 19:01 | 0 | 1 |
| 20:01 | 0 | 1 |
| 28:01 | 1 | 0 |

## Slide 4
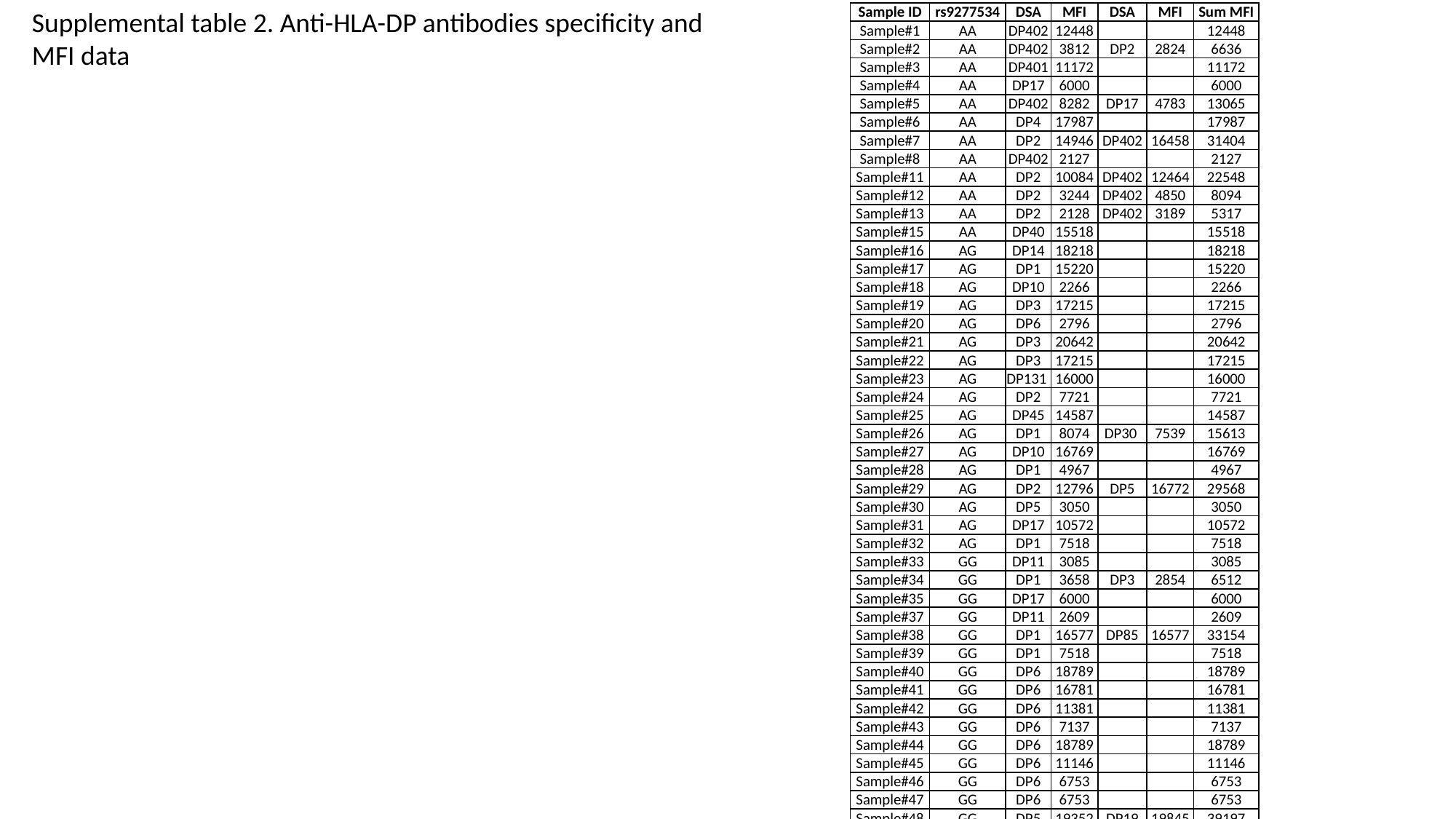

Supplemental table 2. Anti-HLA-DP antibodies specificity and MFI data
| Sample ID | rs9277534 | DSA | MFI | DSA | MFI | Sum MFI |
| --- | --- | --- | --- | --- | --- | --- |
| Sample#1 | AA | DP402 | 12448 | | | 12448 |
| Sample#2 | AA | DP402 | 3812 | DP2 | 2824 | 6636 |
| Sample#3 | AA | DP401 | 11172 | | | 11172 |
| Sample#4 | AA | DP17 | 6000 | | | 6000 |
| Sample#5 | AA | DP402 | 8282 | DP17 | 4783 | 13065 |
| Sample#6 | AA | DP4 | 17987 | | | 17987 |
| Sample#7 | AA | DP2 | 14946 | DP402 | 16458 | 31404 |
| Sample#8 | AA | DP402 | 2127 | | | 2127 |
| Sample#11 | AA | DP2 | 10084 | DP402 | 12464 | 22548 |
| Sample#12 | AA | DP2 | 3244 | DP402 | 4850 | 8094 |
| Sample#13 | AA | DP2 | 2128 | DP402 | 3189 | 5317 |
| Sample#15 | AA | DP40 | 15518 | | | 15518 |
| Sample#16 | AG | DP14 | 18218 | | | 18218 |
| Sample#17 | AG | DP1 | 15220 | | | 15220 |
| Sample#18 | AG | DP10 | 2266 | | | 2266 |
| Sample#19 | AG | DP3 | 17215 | | | 17215 |
| Sample#20 | AG | DP6 | 2796 | | | 2796 |
| Sample#21 | AG | DP3 | 20642 | | | 20642 |
| Sample#22 | AG | DP3 | 17215 | | | 17215 |
| Sample#23 | AG | DP131 | 16000 | | | 16000 |
| Sample#24 | AG | DP2 | 7721 | | | 7721 |
| Sample#25 | AG | DP45 | 14587 | | | 14587 |
| Sample#26 | AG | DP1 | 8074 | DP30 | 7539 | 15613 |
| Sample#27 | AG | DP10 | 16769 | | | 16769 |
| Sample#28 | AG | DP1 | 4967 | | | 4967 |
| Sample#29 | AG | DP2 | 12796 | DP5 | 16772 | 29568 |
| Sample#30 | AG | DP5 | 3050 | | | 3050 |
| Sample#31 | AG | DP17 | 10572 | | | 10572 |
| Sample#32 | AG | DP1 | 7518 | | | 7518 |
| Sample#33 | GG | DP11 | 3085 | | | 3085 |
| Sample#34 | GG | DP1 | 3658 | DP3 | 2854 | 6512 |
| Sample#35 | GG | DP17 | 6000 | | | 6000 |
| Sample#37 | GG | DP11 | 2609 | | | 2609 |
| Sample#38 | GG | DP1 | 16577 | DP85 | 16577 | 33154 |
| Sample#39 | GG | DP1 | 7518 | | | 7518 |
| Sample#40 | GG | DP6 | 18789 | | | 18789 |
| Sample#41 | GG | DP6 | 16781 | | | 16781 |
| Sample#42 | GG | DP6 | 11381 | | | 11381 |
| Sample#43 | GG | DP6 | 7137 | | | 7137 |
| Sample#44 | GG | DP6 | 18789 | | | 18789 |
| Sample#45 | GG | DP6 | 11146 | | | 11146 |
| Sample#46 | GG | DP6 | 6753 | | | 6753 |
| Sample#47 | GG | DP6 | 6753 | | | 6753 |
| Sample#48 | GG | DP5 | 19352 | DP19 | 19845 | 39197 |
| Sample#49 | GG | DP5 | 12178 | DP19 | 13357 | 25535 |
| Sample#50 | GG | DP6 | 19811 | | | 19811 |
| Sample#51 | GG | DP6 | 10831 | | | 10831 |
| Sample#52 | GG | DP6 | 6663 | | | 6663 |
| Sample#53 | GG | DP6 | 4022 | | | 4022 |

## Slide 5
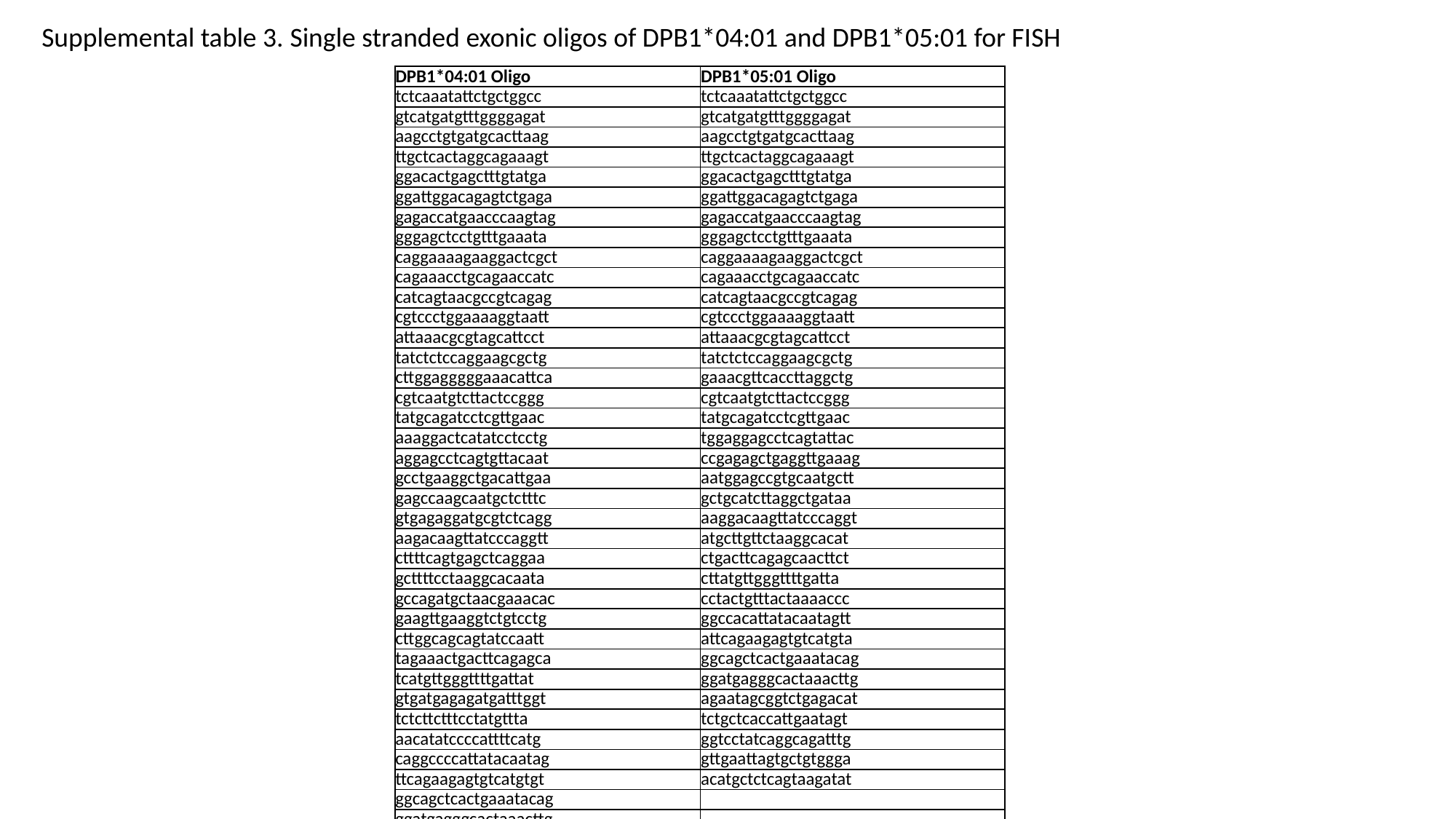

# Supplemental table 3. Single stranded exonic oligos of DPB1*04:01 and DPB1*05:01 for FISH
| DPB1\*04:01 Oligo | DPB1\*05:01 Oligo |
| --- | --- |
| tctcaaatattctgctggcc | tctcaaatattctgctggcc |
| gtcatgatgtttggggagat | gtcatgatgtttggggagat |
| aagcctgtgatgcacttaag | aagcctgtgatgcacttaag |
| ttgctcactaggcagaaagt | ttgctcactaggcagaaagt |
| ggacactgagctttgtatga | ggacactgagctttgtatga |
| ggattggacagagtctgaga | ggattggacagagtctgaga |
| gagaccatgaacccaagtag | gagaccatgaacccaagtag |
| gggagctcctgtttgaaata | gggagctcctgtttgaaata |
| caggaaaagaaggactcgct | caggaaaagaaggactcgct |
| cagaaacctgcagaaccatc | cagaaacctgcagaaccatc |
| catcagtaacgccgtcagag | catcagtaacgccgtcagag |
| cgtccctggaaaaggtaatt | cgtccctggaaaaggtaatt |
| attaaacgcgtagcattcct | attaaacgcgtagcattcct |
| tatctctccaggaagcgctg | tatctctccaggaagcgctg |
| cttggagggggaaacattca | gaaacgttcaccttaggctg |
| cgtcaatgtcttactccggg | cgtcaatgtcttactccggg |
| tatgcagatcctcgttgaac | tatgcagatcctcgttgaac |
| aaaggactcatatcctcctg | tggaggagcctcagtattac |
| aggagcctcagtgttacaat | ccgagagctgaggttgaaag |
| gcctgaaggctgacattgaa | aatggagccgtgcaatgctt |
| gagccaagcaatgctctttc | gctgcatcttaggctgataa |
| gtgagaggatgcgtctcagg | aaggacaagttatcccaggt |
| aagacaagttatcccaggtt | atgcttgttctaaggcacat |
| cttttcagtgagctcaggaa | ctgacttcagagcaacttct |
| gcttttcctaaggcacaata | cttatgttgggttttgatta |
| gccagatgctaacgaaacac | cctactgtttactaaaaccc |
| gaagttgaaggtctgtcctg | ggccacattatacaatagtt |
| cttggcagcagtatccaatt | attcagaagagtgtcatgta |
| tagaaactgacttcagagca | ggcagctcactgaaatacag |
| tcatgttgggttttgattat | ggatgagggcactaaacttg |
| gtgatgagagatgatttggt | agaatagcggtctgagacat |
| tctcttctttcctatgttta | tctgctcaccattgaatagt |
| aacatatccccattttcatg | ggtcctatcaggcagatttg |
| caggccccattatacaatag | gttgaattagtgctgtggga |
| ttcagaagagtgtcatgtgt | acatgctctcagtaagatat |
| ggcagctcactgaaatacag | |
| ggatgagggcactaaacttg | |
| agaatagtggtctgagacat | |
| tctgctcaccattgaatagt | |
| ggtcctatcaggcagatttg | |
| gttgaattagtgctgtggga | |
| acatgctctcagtaaggtat | |

## Slide 6
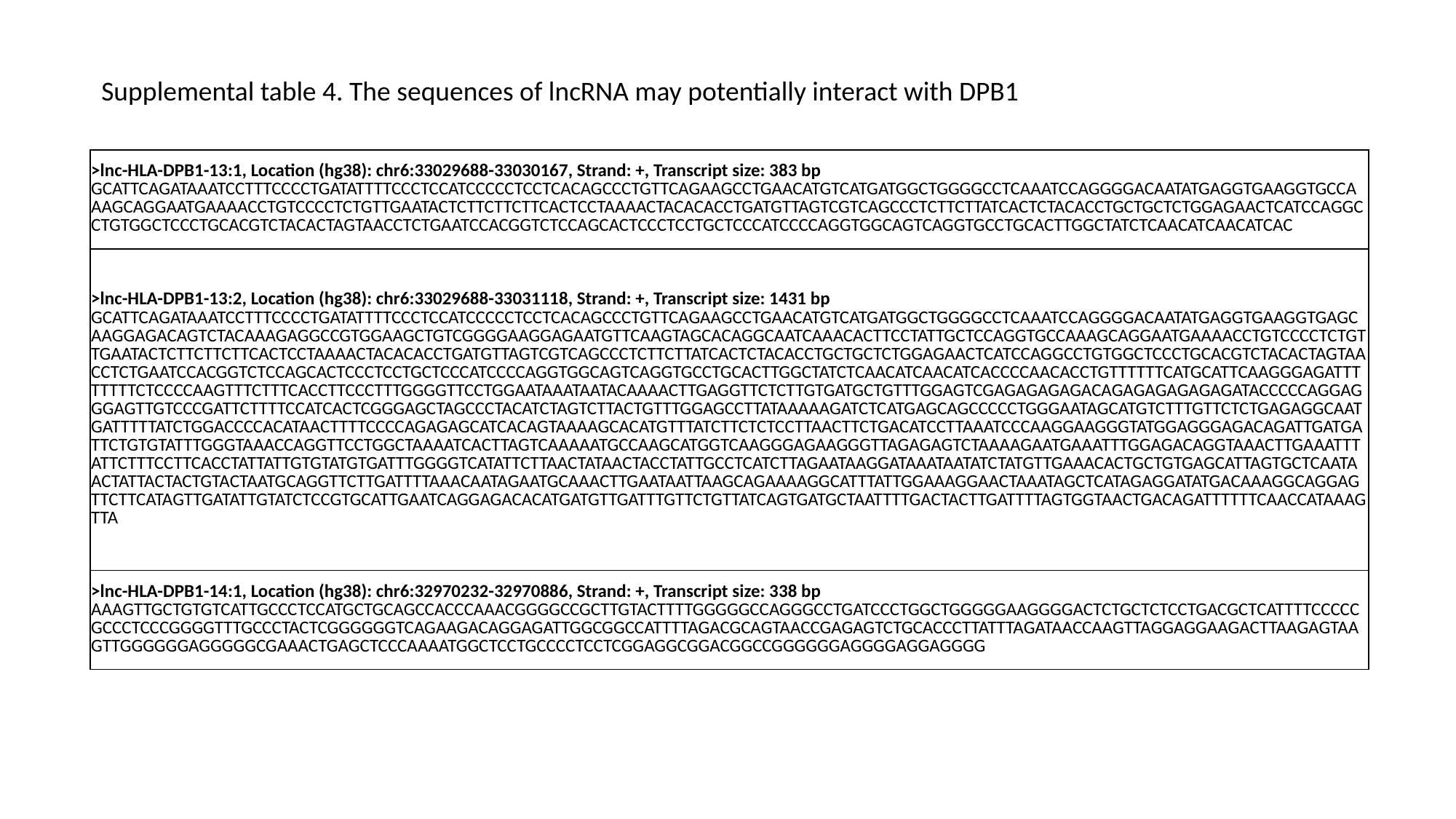

# Supplemental table 4. The sequences of lncRNA may potentially interact with DPB1
| >lnc-HLA-DPB1-13:1, Location (hg38): chr6:33029688-33030167, Strand: +, Transcript size: 383 bp GCATTCAGATAAATCCTTTCCCCTGATATTTTCCCTCCATCCCCCTCCTCACAGCCCTGTTCAGAAGCCTGAACATGTCATGATGGCTGGGGCCTCAAATCCAGGGGACAATATGAGGTGAAGGTGCCAAAGCAGGAATGAAAACCTGTCCCCTCTGTTGAATACTCTTCTTCTTCACTCCTAAAACTACACACCTGATGTTAGTCGTCAGCCCTCTTCTTATCACTCTACACCTGCTGCTCTGGAGAACTCATCCAGGCCTGTGGCTCCCTGCACGTCTACACTAGTAACCTCTGAATCCACGGTCTCCAGCACTCCCTCCTGCTCCCATCCCCAGGTGGCAGTCAGGTGCCTGCACTTGGCTATCTCAACATCAACATCAC |
| --- |
| >lnc-HLA-DPB1-13:2, Location (hg38): chr6:33029688-33031118, Strand: +, Transcript size: 1431 bp GCATTCAGATAAATCCTTTCCCCTGATATTTTCCCTCCATCCCCCTCCTCACAGCCCTGTTCAGAAGCCTGAACATGTCATGATGGCTGGGGCCTCAAATCCAGGGGACAATATGAGGTGAAGGTGAGCAAGGAGACAGTCTACAAAGAGGCCGTGGAAGCTGTCGGGGAAGGAGAATGTTCAAGTAGCACAGGCAATCAAACACTTCCTATTGCTCCAGGTGCCAAAGCAGGAATGAAAACCTGTCCCCTCTGTTGAATACTCTTCTTCTTCACTCCTAAAACTACACACCTGATGTTAGTCGTCAGCCCTCTTCTTATCACTCTACACCTGCTGCTCTGGAGAACTCATCCAGGCCTGTGGCTCCCTGCACGTCTACACTAGTAACCTCTGAATCCACGGTCTCCAGCACTCCCTCCTGCTCCCATCCCCAGGTGGCAGTCAGGTGCCTGCACTTGGCTATCTCAACATCAACATCACCCCAACACCTGTTTTTTCATGCATTCAAGGGAGATTTTTTTTCTCCCCAAGTTTCTTTCACCTTCCCTTTGGGGTTCCTGGAATAAATAATACAAAACTTGAGGTTCTCTTGTGATGCTGTTTGGAGTCGAGAGAGAGACAGAGAGAGAGAGATACCCCCAGGAGGGAGTTGTCCCGATTCTTTTCCATCACTCGGGAGCTAGCCCTACATCTAGTCTTACTGTTTGGAGCCTTATAAAAAGATCTCATGAGCAGCCCCCTGGGAATAGCATGTCTTTGTTCTCTGAGAGGCAATGATTTTTATCTGGACCCCACATAACTTTTCCCCAGAGAGCATCACAGTAAAAGCACATGTTTATCTTCTCTCCTTAACTTCTGACATCCTTAAATCCCAAGGAAGGGTATGGAGGGAGACAGATTGATGATTCTGTGTATTTGGGTAAACCAGGTTCCTGGCTAAAATCACTTAGTCAAAAATGCCAAGCATGGTCAAGGGAGAAGGGTTAGAGAGTCTAAAAGAATGAAATTTGGAGACAGGTAAACTTGAAATTTATTCTTTCCTTCACCTATTATTGTGTATGTGATTTGGGGTCATATTCTTAACTATAACTACCTATTGCCTCATCTTAGAATAAGGATAAATAATATCTATGTTGAAACACTGCTGTGAGCATTAGTGCTCAATAACTATTACTACTGTACTAATGCAGGTTCTTGATTTTAAACAATAGAATGCAAACTTGAATAATTAAGCAGAAAAGGCATTTATTGGAAAGGAACTAAATAGCTCATAGAGGATATGACAAAGGCAGGAGTTCTTCATAGTTGATATTGTATCTCCGTGCATTGAATCAGGAGACACATGATGTTGATTTGTTCTGTTATCAGTGATGCTAATTTTGACTACTTGATTTTAGTGGTAACTGACAGATTTTTTCAACCATAAAGTTA |
| >lnc-HLA-DPB1-14:1, Location (hg38): chr6:32970232-32970886, Strand: +, Transcript size: 338 bp AAAGTTGCTGTGTCATTGCCCTCCATGCTGCAGCCACCCAAACGGGGCCGCTTGTACTTTTGGGGGCCAGGGCCTGATCCCTGGCTGGGGGAAGGGGACTCTGCTCTCCTGACGCTCATTTTCCCCCGCCCTCCCGGGGTTTGCCCTACTCGGGGGGTCAGAAGACAGGAGATTGGCGGCCATTTTAGACGCAGTAACCGAGAGTCTGCACCCTTATTTAGATAACCAAGTTAGGAGGAAGACTTAAGAGTAAGTTGGGGGGAGGGGGCGAAACTGAGCTCCCAAAATGGCTCCTGCCCCTCCTCGGAGGCGGACGGCCGGGGGGAGGGGAGGAGGGG |
